# A Novel Classification Method for Syndrome Differentiation of Patients with AIDS

**DOI:** 10.1155/2015/936290

**Published:** 2015-06-09

**Authors:** Yufeng Zhao, Liyun He, Qi Xie, Guozheng Li, Baoyan Liu, Jian Wang, Xiaoping Zhang, Xiang Zhang, Lin Luo, Kun Li, Xianghong Jing

**Affiliations:** ^1^Institute of Basic Research in Clinical Medicine, China Academy of Chinese Medical Sciences, Beijing 100700, China; ^2^Key Laboratory of Advanced Information Science and Network Technology of Beijing, Beijing Jiaotong University, Beijing 100044, China; ^3^China Academy of Chinese Medical Sciences, Beijing 100700, China; ^4^Institute of Acupuncture and Moxibustion, China Academy of Chinese Medical Sciences, Beijing 100700, China

## Abstract

We consider the analysis of an AIDS dataset where each patient is characterized by a list of symptoms and is labeled with one or more TCM syndromes. The task is to build a classifier that maps symptoms to TCM syndromes. We use the minimum reference set-based multiple instance learning (MRS-MIL) method. The method identifies a list of representative symptoms for each syndrome and builds a Gaussian mixture model based on them. The models for all syndromes are then used for classification via Bayes rule. By relying on a subset of key symptoms for classification, MRS-MIL can produce reliable and high quality classification rules even on datasets with small sample size. On the AIDS dataset, it achieves average precision and recall 0.7736 and 0.7111, respectively. Those are superior to results achieved by alternative methods.

## 1. Introduction

Acquired immune deficiency syndrome (AIDS) is common and extremely harmful to humans. Recently, more and more patients have died of AIDS. AIDS is classified as a plague in traditional Chinese medicine (TCM). Though AIDS is not mentioned in the ancient literatures [1], TCM has been able to clarify the initial basic pathogenesis and evolution of AIDS over 30 years of research [2]. Significant clinical practice [3–14] proved that TCM is better at improving clinical symptoms and quality of life in patients with human immunodeficiency virus (HIV) infection/AIDS clinical symptoms. Moreover, TCM is better at reducing pain and some adverse antiviral reactions for patients with AIDS [3]. TCM plays an important role in the treatment of AIDS. In TCM, treatment based on syndrome differentiation is the basis of clinical assessment and clinical study. However, since TCM usually describes diseases with qualitative and fuzzy quantitative words, there is no clear functional relationship between the symptoms and syndromes [15]. Particularly for AIDS, there are few systematic studies of quantitative syndrome differentiation because of the disease complexity and novelty [4]. Moreover, TCM syndrome differentiation (i.e., patient classification) is a challenging problem as there are no reliable gold standards in TCM research [15]. Therefore, exploring the objective and inherent relationships between the symptoms and syndromes, followed by constructing classification models of syndromes, is a fast developing field.

Currently, some innovative classification techniques are appealable in quantitative syndrome analysis, for example, naïve Bayes, support vector machine (SVM), k nearest neighbor (kNN), and latent structure models [16–23]. Better results have been obtained for several important diseases, for example, coronary heart disease, viral hepatitis, and diabetes. These methods are divided into discriminative [24–28] and generative models [29–33]. Generative models can be developed from discriminative models and are more suitable for missing data, which is a common problem in clinical research. As an integrative method of both discriminative and generative models, multiple instance learning (MIL) obtains the feature representations of homogeneous data using the differences of heterogeneous data. Moreover, since MIL treats the instances in bags as the training samples, MIL is able to describe the feature distribution of classes with small positive labeled classes and the big negative labeled classes. MIL is able to resolve the problem of TCM syndrome differentiation because of two main considerations. On the one hand, the standardization and objectification of TCM syndrome differentiation not only need to identify the syndrome classes of patients, but also give the symptoms representation of each syndrome class. On the other hand, since each patient with AIDS is diagnosed with more than one TCM syndrome, the sample of an AIDS syndrome is smaller than that of all other AIDS syndromes. That is, the positive samples are small and the negative samples are big when a patient is taken as a bag and his symptoms are taken as the instances in the bag. Therefore, MIL is suitable to resolve the problem of TCM syndrome differentiation.

MIL was proposed by Dietterich et al. [34, 35] for the prediction of drug molecule activity. MIL has become widely used in many applications [36–42]. One kind of existing MIL methods embeds each bag into an instance space based on a representative instance set selected from the training bags and then to learn a classifier in the instance space. This kind of method mainly uses the representative instances and similar function to map bags into an instance space, which includes multiple instance learning via embedded instance selection (MILES) [36], diverse density based support vector machine (DD-SVM) [37], key instance detection (KID) [38], multiple instance learning with instance selection (MILIS) [39], multiple instance learning via disambiguation (MILD_B) [40], multiple instance learning via dominant sets (MILDS) [41], and multiple instance learning via constructive covering algorithm (MilCa) [42]. However, it is inappropriate to use these novel MIL methods to resolve the problem of AIDS syndrome differentiation. That is, since the patients with AIDS often experience complications, such as dermatosis, hepatitis, TCM symptoms of intraclass syndromes will be relevant to each other. Some similar symptoms overlap for different AIDS syndromes. In fact, the samples of syndrome differentiation need to be larger if the symptoms of patients with AIDS overlap more. Thus, the symptoms overlap causes the small sample problem for AIDS syndrome differentiation. However, these MIL methods with instance selection ignore the problem of small samples. That is, the learning performance is degraded greatly when less labeled training bags are provided for most existing MIL methods with instance selection. To address the problem of small samples, minimum reference set (MRS) is a prospective method applied to many studies [43]. Therefore, inspired by the above analysis, we attempt to touch on the small sample problem and propose a novel AIDS syndrome differentiation method based on MRS-MIL. With only a small set of labeled patients with AIDS for a syndrome class, the motivation of MRS-MIL is to efficiently select a collection of representative instances (i.e., symptoms) embedded in the positive bags (i.e., patients). Then, the selected representative instances are used to build the feature models of AIDS syndromes. Compared to existing syndrome differentiation methods, the performance of syndrome differentiation based on MRS-MIL is significantly improved even with small samples.

## 2. Materials and Methods

### 2.1. Dataset of AIDS in TCM

The AIDS data comes from the TCM pilot project for treating AIDS, which began in August 2004 in 17 provinces. The ethics committees of Institute of Basic Research in Clinical Medicine, China Academy of Chinese Medical Sciences, granted exempt status for this study and also waived the need for informed consent. Until May 2013, 12,080 patients participated in the project and were treated with Chinese herbs. The treatment is classified into two groups, that is, Chinese herbs and Chinese herbs integrated with Western medicine. The longest treatment of patients with AIDS was continuous 92 months. From the entire AIDS dataset, we selected 3,500 cases based on the inclusion criteria. Inclusion criteria of the patients were (1) age more than 18; (2) TCM syndrome diagnosis record completed; (3) explicit symptoms; (4) patients presenting with at least two symptoms; (5) patients providing informed consent. Of the 3,500 patients, 2,197 patients are male (62.76% with mean age of 38.79 ± 8.02), and 1,303 patients are female (37.24% and 35.67 ± 6.36). The symptoms collected for the case report form (CRF) included a total of 88 symptoms under nine dimensions: skin, chest and abdomen, head, fever, sweating, appetite, arthritis, tongue, and pulse. According to the AIDS syndrome diagnostic standard [44], there are seven syndromes for these 3,500 patients with AIDS. They are (C1) phlegm-heat obstructing the lung and accumulation of heat toxin syndrome; (C2) deficiency of both qi and yin and deficiency of lung and kidney syndrome; (C3) stasis blood and qi deficiency and toxin stasis syndrome; (C4) hot liver and accumulated dampness toxicity syndrome; (C5) stagnation of qi and phlegm and stasis blood syndrome; (C6) deficiency of spleen and stomach and dampness retention syndrome; and (C7) qi deficiency and kidney yin deficiency syndrome.

### 2.2. Methods

**Figure 1:**
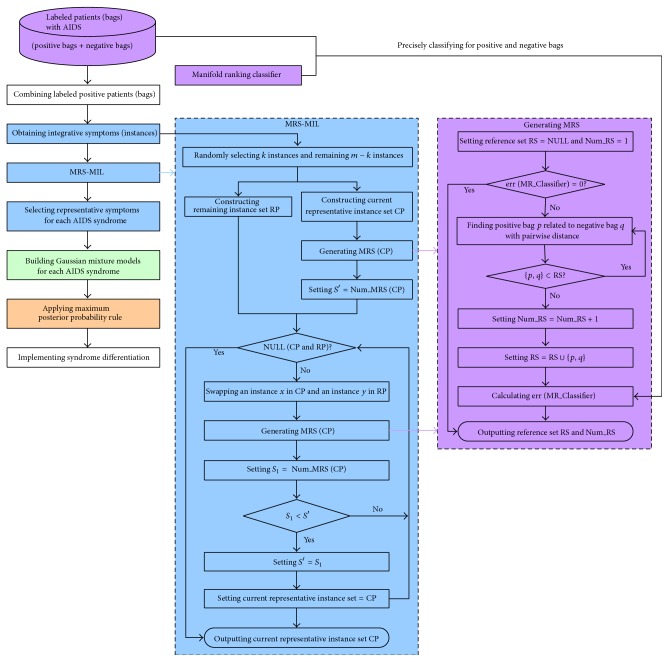
Framework of AIDS syndrome differentiation based on MRS-MIL.

There are three main aspects in AIDS syndrome differentiation based on MRS-MIL. The framework of the proposed method is shown in Figure 1.

First, the representative instances (i.e., symptoms) are selected from the labeled bags (i.e., patients) by MRS-MIL algorithm. Here, a patient is taken as a bag and symptoms are taken as the instances in the bag. A bag is labeled positive as long as one of its instances is positive and a bag is labeled negative only if its instances are all negative. The purpose of MRS-MIL is to find a point with a high density of positive instances and a low density of negative instances. This is identical to finding some symptoms related to a given AIDS syndrome but unrelated to all other AIDS syndromes. Thus, in the process of MIL, these found symptoms are named as the representative instances of the given AIDS syndrome. During the learning process of MRS-MIL in Figure 1, each current representative instance set is associated with its MRS obtained by the algorithm of generating MRS. The size of MRS is a nice measurement for evaluating the significance of representative instance set chosen. Smaller MRS size indicates that fewer labeled bags are selected to build the classifier. That is, the fewer selected labeled bags have better generalization ability to classify the other bags. Thus, the representative instance set appears to be an optimal choice if its size of MRS is the smallest. During the process of generating MRS in Figure 1, MRS denotes the smallest subset of labeled bags that can correctly classify all labeled bags. Starting with an empty set, the reference set is updated by adding the closest bags between two classes until all labeled bags are correctly classified by the algorithm of the manifold ranking (MR) classifier. The generalization of reference set is irrelevant to the number of the labeled training bags. Thus, MRS is especially suitable for the situation of limited labeled training bags. The detailed algorithm is referred to Zhao et al. [45].

Second, based on the selected representative instances, Gaussian mixture model (GMM) is used to characterize the features of the given AIDS syndrome. Here, the parameters of GMM are determined by expectation maximization (EM) and minimum description length (MDL) [46].

Third, AIDS syndrome differentiation is implemented by the rule of maximum posterior probability. The top three AIDS syndromes are selected for the test bag.

## 3. Results

### 3.1. Experimental Method and Evaluation Indicators

In the AIDS dataset, 80 percent of the samples are randomized as the training set and the other 20 percent are chosen as the testing set. The performance of syndrome differentiation based on MRS-MIL is calculated after retesting the models 10 times and taking the mean value. The following criteria are used to evaluate the performance of AIDS syndrome differentiation based on MRS-MIL.

*Precision* evaluates the fraction of syndrome labels ranked above a particular label, which actually is in the label set as Precison = *B*/*A*. The performance is perfect when it is 1; the larger the average precision value, the better the classifier performance.

*Recall* evaluates how difficult it is for the labeled syndrome, on average, to review the list of syndrome labels in order to select all the proper labels of the instance as Recall = *B*/*C*; the performance is perfect when it is 1; the better the recall value, the better the performance.

Here, *A* is the number of patients automatically differentiated with the given AIDS syndrome in the top three of the returned syndrome list; *B* is the number of patients correctly differentiated with that AIDS syndrome in the top three returned syndrome list; and *C* is the number of patients having that syndrome in ground truth syndrome list.

*Selected precision* evaluates the performance of selected representative instances. Referring to the syndrome diagnosis criteria in [44], the selected precision is evaluated as the percentage of compliance between the representative symptoms and the standard symptoms.

*Parameter setting* is as follows. In MRS-MIL, the classification error, which is the end condition of the iteration process of generating MRS, is set to be 0 in order to obtain the best classification result by MR classifier. The parameters (i.e., mean, variance, and weight) of GMM are learnt determined by EM algorithm and the parameter *k* is decided by MDL [46]. The estimation of parameters is an adaptive process.

### 3.2. Selected Representative Symptoms for Each AIDS Syndrome

Syndrome differentiation based on MRS-MIL selects the representative symptoms to characterize the features of each AIDS syndrome.  Table 1 illustrates the selected representative symptoms for seven AIDS syndromes.

### 3.3. Comparison with Other MIL Methods with Representative Instances

To evaluate the performance of MRS-MIL selecting representative instances, we compare it with other relevant MIL methods with representative instances, that is, MILD_B, MILIS, KID, and MilCa.  Table 2 illustrates the compared quality of selected representative instances.

### 3.4. Comparison with Other Syndrome Differentiation Methods

Currently, the common data mining methods are directly used for TCM syndrome differentiation. This is the main reason for unsatisfied results of syndrome differentiation. In this paper, we propose a novel MRS-MIL classification method to specifically be used for TCM syndrome differentiation of patients with AIDS. To evaluate the performance of syndrome differentiation based on MRS-MIL, we compare it to existing syndrome differentiation methods: *k*-mean, naïve Bayes, and SVM.  Table 3 illustrates the comparative results.

### 3.5. Sensitivity to the Number of Labeled Patients

**Figure 2:**
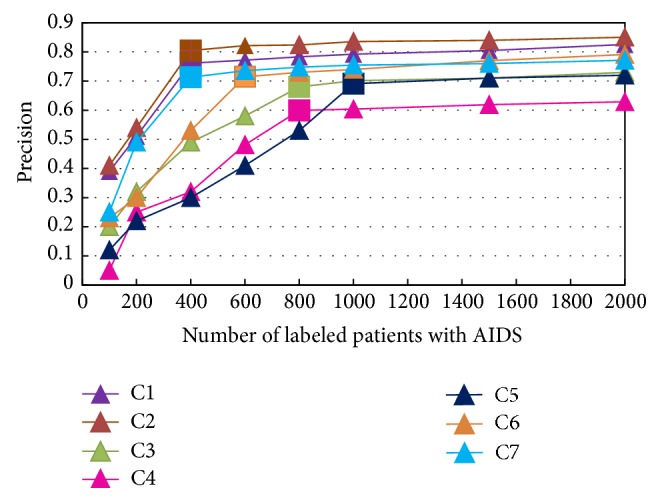
Influence of various numbers of labeled patients with AIDS.

To further investigate the advantages of MRS-MIL with smaller labeled bags, we show the precision of seven AIDS syndromes. By gradually increasing the number of labeled patients, Figure 2 illustrates the average precision curve for each AIDS syndrome. As given, there are 100, 200, 400, 600, 800, 1,000, 1,500, and 2,000 labeled patients with AIDS used to calculate the performance of syndrome differentiation based on MRS-MIL. Note that the labeled patients of each AIDS syndrome are randomly selected from the experimental dataset.

### 3.6. MR versus 1-NN for Generating MRS

**Figure 3:**
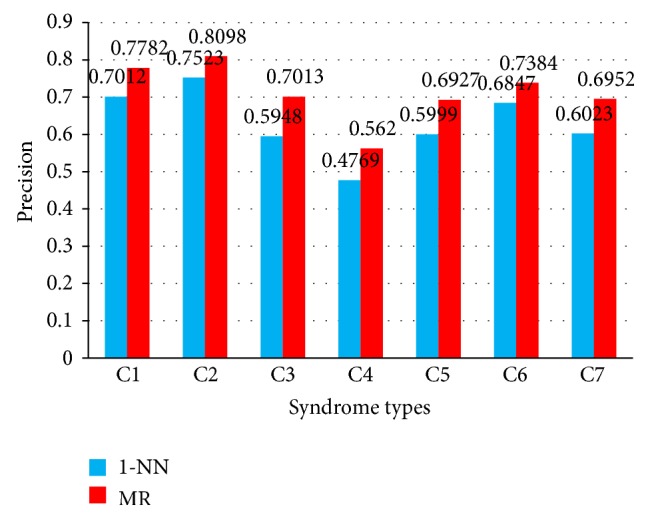
Recall of seven syndromes.

According to the selection algorithm in Methods, the representative instances embedded in the positive bags are more precise if the generated MRS is more accurate. Thus, it is significant for generating better MRS. Instead of applying the 1-NN classifier, MRS is generated via the MR classifier in this paper. The MR classifier can benefit from the intrinsic global structure revealed by the labeled and unlabeled bags. That is, the intrinsic global structure is not relevant to the number of labeled training bags. To show the advantages of MRS generated by the MR classifier, Figure 3 demonstrates the recall of seven AIDS syndromes with 3,500 samples.

## 4. Discussion

### 4.1. Visual Symptoms Representation of AIDS Syndromes

According to Table 1, MRS-MIL selects the representative symptoms to characterize the feature of each AIDS syndrome well. In detail, the C1 syndrome and C2 syndrome obtain more than 90% selected precision. In particular, the selected precision of the C2 syndrome is best, reaching 0.9233. Moreover, even for the worst performance (C4 syndrome), the selection precision is achieved at 0.8079. In addition, for the seven AIDS syndromes, the average selected precision of representative symptoms is more than 80% by the AIDS clinical experts' evaluation. The results show the high common viewpoint for the clinical experts. By the selected representative symptoms of each syndrome, patients with AIDS can also be automatically diagnosed and the experience of famous experts can also be subsumed in TCM clinical practice. We can conclude that some representative symptoms characterize the feature of each AIDS syndrome by MRS-MIL well.

### 4.2. Selected Representative Instances Performance

From the results in Table 2, the average selected precision of MRS-MIL is the best. In detail, the average selected precision of the seven AIDS syndromes is 0.6676, 0.7243, 0.7684, 0.8148, and 0.8568 for MILD_B, MILIS, KID, MilCa, and MRS-MIL, respectively. Compared to the other MIL methods with representative instances, MRS-MIL gains 18.93%, 13.25%, 8.84%, and 4.20% improvement, respectively. The best selected precision of MILD_B and MILIS is achieved for C6 syndrome and C7 syndrome, respectively. The best precision of KID, MilCa, and MRS-MIL is all achieved at C2 syndrome. We can conclude that the performance of MRS-MIL selecting representative instances is better than state-of-the-art MIL with representative instances.

### 4.3. Syndrome Differentiation Performance

As shown in Table 3, the results of syndrome differentiation based on MRS-MIL obtain better performance than *k*-means, naïve Bayes, and SVM. The SVM results are better than the Bayesian. The *k*-means method has the worst performance. In detail, the average precision of syndrome differentiation based on MRS-MIL is 0.7736 and the average recall is 0.7111. Compared to *k*-means, Bayesian, and SVM, MRS-MIL gains 21.08%, 22.33%, and 27.76% improvement, respectively, for the average precision of seven AIDS syndromes. The best precision of *k*-means, Bayesian, and SVM is achieved at C1 syndrome. The best precision of MRS-MIL is achieved at C2 syndrome. Similarly, compared to *k*-means, naïve Bayes, and SVM, MRS-MIL is able to gain 24.79%, 22.04%, and 27.54%, respectively, for the average recall of seven AIDS syndromes. The best recall of *k*-means is achieved at C6 syndrome. The best recall of Bayesian and SVM is achieved at C1 syndrome. The best recall of MRS-MIL is achieved at C2 syndrome. We conclude that the MRS-MIL performance of syndrome differentiation is better than existing syndrome differentiation methods.

### 4.4. Influence of Small Samples

From the trend of cures in Figure 2, the precision of syndrome differentiation is insensitive to the small samples of seven AIDS syndromes. The precision of syndrome differentiation based on MRS-MIL maintains a steady status when the samples reach a specific number of samples. Obviously, there is a steady status for all the AIDS syndromes. In Figure 2, the bigger square in each cure denotes the steady point. The final precision of syndrome differentiation is reached at 0.8504, 0.8256, 0.7300, 0.6289, 0.7200, 0.7911, and 0.7712 for the seven AIDS syndromes, respectively. However, the precision of syndrome differentiation improves slightly when the samples of seven AIDS syndromes arrive at 400, 400, 800, 800, 1,000, 600, and 400, respectively. In detail, C1, C2, C3, C4, C5, C6, and C7 syndromes will be steady at (400, 0.8047), (400, 0.7614), (800, 0.6801), (800, 0.5993), (1,000, 0.6911), (600, 0.7147), and (400, 0.7132), respectively. That is, for seven AIDS syndromes, the precision of syndrome differentiation only improves slightly (4.57%, 6.42%, 4.99%, 2.97%, 2.89%, 7.64%, and 5.8%) with a significant increase in number of labeled patients (1,600, 1,600, 1,600, 1,200, 1,200, 1,000, 1,400, 1,400, and 1,600). We conclude that the performance of syndrome differentiation based on MRS-MIL is still good even with small samples.

### 4.5. MR versus 1-NN for Generating MRS

As shown in Figure 3, it is obvious that MR based performance is better than 1-NN, since the precision is improved from 75.23% to 80.98% for C2 syndrome and the precision is boosted by 5.37% even for C6 syndrome. In particular, the average precision is promoted by 8.08% for total seven AIDS syndromes. In detail, the recall based on MR classifier gains the most improvement (10.65%) against 1-NN classifier for C3 syndrome and the least improvement (5.37%) for C6 syndrome. For C1, C2, C4, C5, and C7 syndromes, the recall based on MR classifier gains 7.70%, 5.75%, 8.51%, 9.28%, and 9.29% improvement, respectively, against the 1-NN classifier. We conclude that the performance based on MR classifier is better than that based on 1-NN classifier.

## 5. Conclusions

MRS-MIL-based classification methods facilitated the building of syndrome differentiation models for patients with AIDS. Syndrome differentiation based on MRS-MIL can not only select the representative instances for each AIDS syndrome, but also provide a practical solution to the small sample problem. Compared to other classification methods, this method improves the average syndrome differentiation precision of seven AIDS syndromes as well as the average syndrome differentiation recall. MRS-MIL also improves average selected precision of representative instances compared to the state-of-the-art MIL methods with representative instances.

There are three advantages of syndrome differentiation based on MRS-MIL. First, compared to the discriminant syndrome differentiation methods, syndrome differentiation based on MRS-MIL can accurately select the representative symptoms to explicitly characterize the features of AIDS syndromes. This will provide reliable evidence for the standardization and objectification of TCM syndrome differentiation. Moreover, since TCM development is a method of empirical medicine, the method of explicit representative symptoms for TCM syndrome is a feasible way to propagate the experience of famous TCM experts. Second, syndrome differentiation based on MRS-MIL can gain good performance even using small samples for patients with AIDS. On the one hand, each patient usually has several AIDS syndromes because of disease complexity. There are some similar symptoms for different AIDS syndromes. The samples labeled with a special syndrome will become relatively small. On the other hand, methods based on small samples are frequently met in clinical research since collecting clinical cases is difficult and costly. Therefore, compared to the state-of-the-art MIL methods with representative instances, syndrome differentiation based on MRS-MIL has better performance even with small samples and hence is more suitable for TCM clinical study. Third, the intrinsic global structure of AIDS symptoms can be revealed by the MR classifier well. Hence, the performance of syndrome differentiation based on MRS-MIL is improved as per the experimental results.

The disadvantage of MRS-MIL is that the selected symptoms have the same representative degree for each AIDS syndrome. In the future, MRS-MIL will address the representative degree of selected symptoms to AIDS syndromes.

## Figures and Tables

**Table 1:** Selected representative symptoms for seven AIDS syndromes.

AIDS syndrome	Selected representative symptoms	Precision
C1	White fur; string-like pulse; fever; rash or herpes; red tongue; dizziness; chest pain; insomnia; cough	0.9026

C2	Loose stool; fatigue; night sweats; pale complexion; red tongue; sticky sputum; low fever; rapid pulse; blood clots; yellow urine; skin ulcer	0.9233

C3	Dry mouth; dyspnea on exertion; stationary pain; local fever of body; loose stool; dark and gloomy complexion; small blister; fever in the afternoon and at night; white fur; alopecia; deep pulse; dark purple tongue	0.8512

C4	Skin ulcer; irritability; herpes; skin itching; red tongue; brief yellow urine; loose stools; asthma; blister searing; slippery pulse	0.8079

C5	Cold sweat; pink tongue; thin fur; depression; string-like pulse; skin itching; lack of appetite; scrofula bump; difficult stool; weight loss	0.8145

C6	Prolapse; loose stools; blister searing; diarrhea; nausea; deep pulse; tired soreness; greasy fur; abdominal pain; slippery pulse; anal burning; diarrhea; pharyngeal	0.8324

C7	Fatigue; diarrhea; dry mouth; fever; chills; grey fur; difficult stool; tired soreness; blister searing; weak pulse	0.8658

**Table 2:** Precision of selected representative instances of various MIL methods.

AIDS syndromes	Precision of selected representative instances				
	MILD_B	MILIS	KID	MilCa	MRS-MIL
C1	0.6713	0.7361	0.7707	0.8541	0.9026
C2	0.7026	0.7699	**0.8216**	**0.8832**	**0.9233**
C3	0.6436	0.67921	0.7931	0.8046	0.8512
C4	0.6513	0.6613	0.7156	0.7681	0.8079
C5	0.5925	0.6984	0.7654	0.7934	0.8145
C6	**0.7135**	0.7518	0.7116	0.7769	0.8324
C7	0.6981	**0.7735**	0.8011	0.8234	0.8658

Avg.	**0.6676**	**0.7243**	**0.7684**	**0.8148**	**0.8568**

**Table 3:** Comparative results of existing syndrome differentiation methods.

AIDS syndromes	Syndrome differentiation methods							
	*k*-means		Naïve Bayes		SVM		MRS-MIL	
	Precision	Recall	Precision	Recall	Precision	Recall	Precision	Recall
C1	**0.5133**	0.4435	**0.6236**	**0.5600**	**0.6255**	**0.5529**	0.8327	0.7782
C2	0.5089	0.4512	0.5592	0.5029	0.5913	0.4639	**0.8526**	**0.8098**
C3	0.5001	0.4359	0.5208	0.4756	0.5347	0.4378	0.7771	0.7013
C4	0.4642	0.3815	0.4714	0.4011	0.4923	0.3874	0.6303	0.5620
C5	0.4912	0.4209	0.5108	0.4398	0.5488	0.4711	0.7371	0.6927
C6	0.5024	**0.4711**	0.5988	0.5219	0.5835	0.4826	0.8012	0.7384
C7	0.4920	0.4456	0.5678	0.5333	0.6011	0.4465	0.7843	0.6952

Avg.	**0.4960**	**0.4357**	**0.5503**	**0.4907**	**0.5682**	**0.4632**	**0.7736**	**0.7111**
